# How Individuals With Down Syndrome Process Faces and Words Conveying Emotions? Evidence From a Priming Paradigm

**DOI:** 10.3389/fpsyg.2020.00692

**Published:** 2020-04-17

**Authors:** Maja Roch, Francesca Pesciarelli, Irene Leo

**Affiliations:** ^1^Department of Developmental Psychology and Socialization, University of Padua, Padua, Italy; ^2^Department of Biomedical, Metabolic and Neural Sciences, University of Modena and Reggio Emilia, Modena, Italy; ^3^Center for Neuroscience and Neurotechnology, University of Modena and Reggio Emilia, Modena, Italy

**Keywords:** down syndrome, face perception, priming, emotion recognition, emotion labels

## Abstract

Emotion recognition from facial expressions and words conveying emotions is considered crucial for the development of interpersonal relations (Pochon and Declercq, 2013). Although Down syndrome (DS) has received growing attention in the last two decades, emotional development has remained underexplored, perhaps because of the stereotype of high sociability in persons with DS. Yet recently, there is some literature that is suggesting the existence of specific deficits in emotion recognition in DS. The current study aimed to expand our knowledge on how individuals with DS process emotion expressions from faces and words by adopting a powerful methodological paradigm, namely priming. The purpose is to analyse to what extent emotion recognition in DS can occur through different processes than in typical development. Individuals with DS (*N* = 20) were matched to a control group (*N* = 20) on vocabulary knowledge (PPTV) and non-verbal ability (Raven’s matrices). Subsequently a priming paradigm was adopted: stimuli were photos of faces with different facial expressions (happy, sad, neutral) and three words (happy, sad, neutral). On a computer screen the first item (face or word) was presented for a very short time (prime) and afterward a stimulus (face or word) appeared (target). Participants had to recognize whether the target was an emotion (sad/happy) or not (neutral). Four prime-target pairs were presented (face-word; word-face; word-word; face-word) in two conditions: congruent (same emotion prime/target) and incongruent (different emotion prime/target). The results failed to show evidence for differential processing during emotion recognition between the two groups matched for verbal and non-verbal abilities. Both groups showed a typical priming effect: In the incongruent condition, slower reaction times were recorded, in particular when the target to be recognized is the face, providing evidence that the stimuli were indeed processed. Overall, the data of the current work seem to support the idea of similar developmental trajectories in individuals with DS and TD of the same verbal and non-verbal level, at least as far as the processing of simple visual and linguistic stimuli conveying basic emotions is concerned. Results are interpreted in relation to recent finding on emotion recognition from faces and words in DS.

## Introduction

Down syndrome (DS) is the most common genetic intellectual disability and for this reason, for many years, DS has received great attention from researchers. There are a large number of studies concerning the cognitive and linguistic profile of DS, which is extensively described for this population (Roizen and Patterson, 2003; Abbeduto et al., 2007; Grieco et al., 2015; McDuffie et al., 2017; Lanfranchi, 2019; Roch et al., 2019). Yet, the socio-emotional profile of individuals with DS has remained underexplored. The current study aims to expand our knowledge of emotional skills in young adults with DS through the analysis of the ability to recognize emotional expression and emotion labels adopting a very powerful paradigm, namely, a priming paradigm. This paradigm has never been used for investigating emotion recognition in individuals with DS.

The ability to decode the facial expression of others is considered crucial for the socioemotional competence and for the construction of social interactions and relationships (Calder et al., 2000; Wishart and Pitcairn, 2000; Izard et al., 2001; Denham and Weissberg, 2004; Pochon and Declercq, 2014). The identification of the socioemotional profile is therefore essential in order to provide educational and psychological support. One of the reasons for which this field has attracted only limited research in the population with DS, is related to the stereotype of high sociability in persons with DS (Pitcairn and Wishart, 1994): in fact, they are generally considered proficient at establishing social relationships with others. In addition, the first studies that have investigated emotion competence in DS have found that emotion recognition was in line with their level of intellectual disability.

Turk and Cornish (1998) reported no differences between children with DS and typically developing (TD) children, matched for mental age, in the recognition of facial expressions of happiness, sadness, anger, and fear, as well as in the recognition of emotional vocalizations. Nevertheless, children with DS performed worse than TD children in matching the appropriate facial expression to the context: this result was interpreted as an indication of an impairment in attributing the deep meaning to the emotion. Similarly, Celani et al. (1999) compared the performance of children with DS and TD matched for verbal mental age at an emotion matching task. Children with DS presented similar abilities to the TD children in recognizing the expressions of happiness and sadness but also in rating the valence of emotional expressions and situations, but showed a specific difficulty in identifying anger. Following from this, it was suggested that there might be qualitative differences in socio-emotional functioning between individuals with DS and TD children of the same cognitive level (Cebula and Wishart, 2008; Cebula et al., 2010). Some of these deficits have been, indeed, identified in infancy and childhood and this could have had a negative impact on the subsequent development of interpersonal relationships. It is relevant to adopt a developmental perspective when examining individuals with atypical development, being aware that what we observe in one moment is a result of a developmental process that have had cascading effects on this result (Ewing et al., 2017).

Some recent studies have found emotional difficulties in children and adolescents with DS (Fidler et al., 2008; Jahromi et al., 2008; Martinez-Castilla et al., 2015; Goldman et al., 2017). These works noted weaknesses in emotion recognition of individuals with DS which cannot be explained by their level of cognitive ability (Wishart and Pitcairn, 2000; Williams et al., 2005; Wishart et al., 2007; Cebula et al., 2017). Wishart and Pitcairn (2000) compared a group of individuals with DS to two control groups matched for the level of cognitive ability: one group was composed by children with typical development and the other was composed by individuals with non-specified intellectual disability (ID). Children with DS performed significantly worse when compared to the TD group on a facial expression matching task involving six primary emotions (happiness, sadness, surprise, fear, anger, and disgust); they had particular difficulty in distinguishing fear and surprise. The results of the children with non-specified ID were not different from those of the TD children. Therefore, the impaired performance of DS individuals in this task cannot be attributed to intellectual disability and was, instead attributed to specific features of DS. In line with this interpretation, Williams et al. (2005) hypothesized that there might be a specific impairment of emotion recognition in DS.

Several studies noted that the difficulty that individuals with DS experience regarding the recognition of facial expressions was not generalized to all emotions but was particularly evident for specific emotions, namely, fear (see also Wishart et al., 2007). More generally, Porter et al. (2007) showed that individuals with DS show difficulties in recognizing negative emotions. In addition, Kasari et al. (2001) highlighted that children with DS tend to choose positive expressions instead of negative ones and vice versa. Finally, other atypical errors were highlighted by Williams et al. (2005) and Wishart et al. (2007): they reported that children with DS tended to confuse fear with sadness.

The studies presented in this literature review all adopted methods which minimized the use of language which is controlled for through statystical approaches, for the sake of the linguistic level of participants with DS. Nevertheless, the instructions always involved the understanding of emotional labels. This might have an impact on the performance of individuals with DS because of the influence of emotional language on the emotion perception (Lindquist et al., 2006; Barrett et al., 2007). In this respect, Channell et al. (2014) examined emotion recognition of a group of children and adolescents with DS through tasks that measured the ability to recognize others’ emotions from static and dynamic facial expressions and from the social context and compared to typically developing (TD) children of similar developmental levels. In this study, a measure of emotion recognition that minimized the need for linguistic skills was used. The results indicated similar accuracy for participants with DS and TD participants when judging emotions from static or dynamic expression stimuli and from facial or contextual cues. In a further study, the authors compared the two groups on the rate at which their emotion recognition grew relative to their cognitive level, and EXAMINED the relationship between emotion recognition and developmental factors (mental and chronological age). The results indicated that participants with DS and TD showed similar cross-sectional developmental trajectories of emotion recognition in relation to their mental age and that emotion recognition was correlated to both mental and chronological age in each of the two groups. These two studies showed clearly that, when the use of language skills is reduced in emotion recognition tasks, DS individuals do not differ from TD children in emotion recognition supporting the idea of similarity between groups in this skill relative to the level of cognitive development.

Other recent studies used non-verbal tasks for the analysis of emotion recognition in DS. Pochon and Declercq (2013) conducted a longitudinal study using a non-verbal task for emotion recognition. Children with DS were compared to a group of children with non-specified intellectual disability and TD children matched for non-verbal cognitive ability. The three groups performed a task that required the recognition of six basic emotions: they were asked to match an emotional auditory stimulus (a vocalization) with an emotional visual stimulus (a facial expression). The results revealed similar performance in emotion recognition of the DS group compared to the other two groups of participants. Because of the strictly non-verbal design of this study, this result was interpreted as an indication of possible negative influence of the emotional lexicon when participants show impaired recognition of emotion expressions (Pochon and Declercq, 2013). The results of this study were replicated in a subsequent work with the same participants: the ability to recognize basic emotional facial expressions by means of a non-verbal protocol that uses video clips rather than static photographs was adopted (Pochon and Declercq, 2014). Finally, in a recent study, Pochon et al. (2017), reported new evidence on the absence of differences in emotion recognition between DS and TD matched for non-verbal ability. The authors conclude by highlighting the importance of using dynamic, strictly non-verbal tasks for participants with DS, and more generally for populations with language disorders.

These four studies are the only recent studies in which children with DS did not show weakness in recognizing emotions from facial expressions; nevertheless, they have raised the question of whether the difficulties reported for DS children in previous studies were at least in part due to the use of emotional labels. Notably, individuals with DS are characterized by severe language impairments and emotional lexicon was repeatedly mentioned as one of the weaknesses of their linguistic profile (Chapman, 1997; Chapman and Hesketh, 2000). It is possible then, that even when language level is controlled for, a deficit in emotional vocabulary may disadvantage individuals with DS in emotion recognition tasks. This interpretation would be coherent with some observations that children with Down syndrome are exposed to less conversation about emotional terminology than typically developing children are. Since children with DS tend to be perceived stereotypically as friendly and happy, their caregivers tend to use fewer negative emotion words with them, providing children with reduced opportunities to learn emotions and to match correctly the emotion labels to specific emotions (Tingley et al., 1994; Kasari et al., 2001).

The purpose, therefore, becomes to investigate more deeply the nature of the deficits in emotion recognition of individuals with DS in order to be able to better interpret the results obtained in previous studies.

Most of the literature review reported in the previous paragraphs have involved children and adolescents with DS in their works. However, there are some studies of emotion recognition in adults with DS (Porter and Coltheart, 2006; Hippolyte et al., 2008, 2009; Carvajal et al., 2012; Virji-Babul et al., 2012). For the purposes of the current work, the most relevant data come from the study of Carvajal et al. (2012), in which they showed that adults with DS did not present any specific deficits in matching emotional significance to faces compared to people with general intellectual disability. However, they found that people with DS showed some specific deficits in the first phases of the processing of faces, namely in configuring facial traits. This calls for further research of the first phases in the processing of the faces preceding emotion recognition.

We adopted a specific approach in order to investigate emotion recognition and, in addition, we measured emotion recognition by adopting an implicit recognition approach, namely a priming paradigm in which we minimized the use of emotion labels.

In priming experiments, participants are usually presented with pairs of items displaced in time, a single prime word presented for several milliseconds followed by a single target word. Participants are required to make a response to the target (e.g., naming the target aloud). Priming is measured by comparing response time and accuracy across related and unrelated trials. Numerous studies have shown that processing of the target can be greatly influenced by the nature of the relationship between the prime and target stimuli. Target stimuli are typically associated with faster response times (RTs) and fewer errors when they follow an identity (e.g., dog-dog) or semantically (e.g., cat-dog) related prime relative to when they follow a semantically unrelated (e.g., table-dog) stimulus (Neely, 1991, 1997; McNamara, 1992; Masson, 1995; Pesciarelli et al., 2007). Priming is obtained when response times are faster and/or accuracies are greater for related trials, relative to unrelated trials. The priming effect is remarkably robust and has been observed within a wide variety of experimental settings (see Neely, 1991 for a review). Several mechanisms have been proposed to explain this effect. One which is widely accepted is the automatic spreading activation mechanism. It has been argued that faster response times to related targets are the result of the target already being partially activated by spreading activation from its related prime occurring before target presentation (Neely, 1997).

The priming procedure is increasingly being used as a tool to investigate cognitive mechanisms underlying language, perception, memory, attention and emotion processing. Therefore, this paradigm is well suited to investigate the ability of individuals with Down syndrome to recognize whether a face/label corresponds to an emotion or not.

The existing literature reports evidence on a possible specific impairment of emotion recognition in individuals with DS. At the same time, some studies pointed out that this impairment might reflect a difficulty with emotion labeling that influence the perception and the recognition of emotions and facial expressions (Vicari et al., 2000; Lindquist and Gendron, 2013). The aim of our study is to contribute to a better understanding of the processes involved in the recognition of emotional facial expressions by individuals with DS. To the best of our knowledge, this is the first study that adopted a priming task in order to investigate emotion recognition by individuals with DS. They were compared to a group of typically developing first graders matched for verbal and non-verbal skills. The two groups were required to recognize emotion from facial expressions (photographs) and from emotion labels (written words) through a priming paradigm by distinguishing emotional states from neutral ones. The current work is exploratory rather than confirmatory in nature since, to the best of our knowledge, it is the first time a similar paradigm is adopted for investigating emotion recognition in DS. For this reason, it is not possible to formulate specific predictions regarding the performance of the participants. Nonetheless, the study was designed in order to address four issues for which there is still an open debate in literature on emotion recognition in DS. In particular, the current study was designed:

– To analyse group differences in the ability to distinguish emotions from neutral states.
– To analyse whether the three emotion categories (sad, happy and neutral) are processed differently.
– To analyse whether emotion recognition differs when the emotions have to be identified through words or faces.
– To analyse whether the typical congruency effect expected in the priming paradigm is more pronounced in specific conditions, for specific targets and differs across the two groups.
– Both the accuracy and the reaction times were analyzed through the analyses of variance.

## Materials and Methods

### Participants

Participants consisted of 20 individuals with DS (6 female; chronological mean age = 23 years 3 months, age range = 17–37, SD = 2 years 5 months) and 20 children with typical development (TD) (10 female; chronological mean age = 6 years 5 months, age range = 6–7 years, SD = 2 months). Italian was the native language of all participants. All participants were residents in the province of Padova, Veneto (Italy). Participants with DS came from the Association Down DADI Padova. All of them were able to read. Families and practitioners reported that none of the study participants had symptoms of cognitive decline at the time of the study. All participants with DS are active in the community, they are either attending autonomy courses, working or attending school: these data help us to support the idea that the results we obtain in this work cannot have been influenced by a possible symptomatology of cognitive or memory decline.

The sample of TD were selected from a larger group of 65 participants and were selected on the basis of the following criteria: they were all first graders (they have had to be able to read single words) and were matched to participants with DS on the basis of their score on PPVT (Peabody Picture Vocabulary Test, Dunn and Dunn, 1981; Stella et al., 2000) and on the basis of non-verbal intelligence scales (Coloured Progressive Matrices CPM: Italian version – Belacchi et al., 2008). This allowed us to have a double matching, both on verbal ability (verbal matching = raw score -/+ 5 points) and non-verbal ability (non-verbal matching = raw score -/+ 3 points). Individuals with DS scored on average 96.95 (SD = 22.04) at the PPVT, while TD children scored on average 95.05 (SD = 16.40). The two groups scored at the non-verbal task 14.60 (SD = 3.55) for individuals with DS and 18.55 (SD = 4.20) for TD children. The two groups did not differ either on the measure of PPVT and on CPM [*t* (19) = 0.987, *p* = 0.496 and *t* (19) = 1.2, *p* = 0.193, respectively for PPVT and CPM].

### Stimuli

**FIGURE 1:**
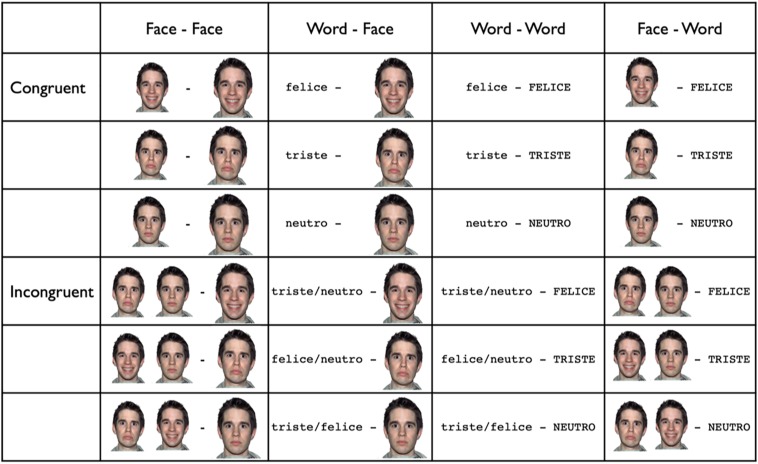
Research design: experimental stimuli.

Three color pictures of real faces (sad, happy, neutral) selected from the NimStim face stimulus set (Tottenham et al., 2009)^1^ and three black letter words (triste-sad, felice-happy, neutro-neutral) were used. The background was white and the mean luminance was approximately the same for all pictures. The prime and the target were either a word or a picture of a real face. Four types of prime-target presentation pairs were used: 1. word-face; 2. word-word; 3. face-face; 4. face-word. The pairs of stimuli (prime-target) in each of the four different presentation types belonged, in half of the trials, to the same emotion (congruent condition) and, in the other half to different emotions (incongruent condition) (see Figure 1). In order to avoid orthographic overlap, prime words were presented in lowercase letters and target words in uppercase letters (Courier font, size 20). Prime faces were 25% smaller (visual angle 8.5°) than target faces (visual angle 11.3°) to avoid any apparent movement between the prime and target stimuli.

Participants performed four blocks of 80 trials each (one word-face; one word-word; one face-face; one face-word) in a total of 320 trials. In each block, the emotion and the prime-target congruency were fully crossed and counterbalanced.

### Design and Procedure

**FIGURE 2:**
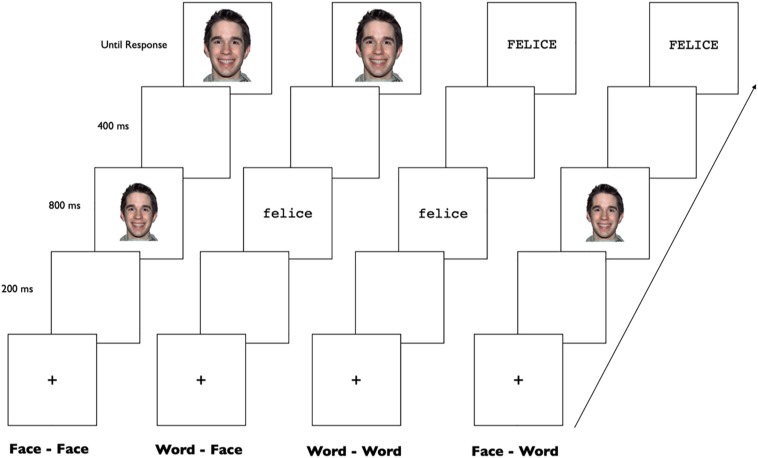
Experimental procedure.

The stimulus presentation procedure is graphically reported in Figure 2. All stimuli (faces and words) were displayed in the center of a CRT monitor synchronous with the screen refresh [refresh rate = 60 Hz (16.67 ms)] that was positioned at eye level approximately 70 cm in front of the participant. E-Prime software (Version 1; Psychology Software Tools, Pittsburgh, PA) was used for stimulus presentation and behavioral response collection. Each trial began with a 1000 ms fixation cross (+) presented in the middle of the screen. Then a white screen was displayed for 200 ms and replaced by a 800 prime stimulus. The prime was then immediately followed by a 400 ms white screen. Then the target appeared and remained on the screen until a response was made. Each response was followed by a 500 ms white screen. The task of the participants was to decide, as quickly and accurately as possible, whether the target represented an emotion or a neutral stimulus. Participants responded by pressing one of two buttons, which were counterbalanced (left and right) across subjects. The participant’s responses controlled the onset and termination of the target on the screen. When the participant pressed one of the response buttons, the stimulus was erased from the screen. Before the experiment, participants took part in a short training session with 12 prime-target pairs (3 for each presentation type). Further, they were asked to read the three words (sad, happy and neutral) and to recognize the three faces. All participants were able to label the three words and the three facial expressions correctly. The task and the procedure were specifically developed for this work therefore, we will provide preliminary data on adopting the priming paradigm in DS, and future works should be designed with a similar procedure in order to provide for additional validation of this paradigm.

### Statistical Analysis

The original dataset consisted of 12800 observations (i.e., 40 subjects × 360 observations) on each of our dependent variables: Accuracy and Reaction Time.

Given the type of experiment, to exclude unreliable observations, we first eliminated all observations with Response Time below 200 ms (i.e., anticipatory responses) and above 3500 ms (i.e., late responses). Specifically, 52 observations with Reaction Time below 200 ms (0.04%) and 708 with Reaction Time above 3500 ms (5.5%) were excluded. It is important to note that the percentage of excluded observations were homogeneous among Down (i.e., 5.61%) and Control (6.27%) groups. Thus, the final dataset for the analysis of Accuracy consisted of 12040 observations.

The analyses on Reaction Times (RTs) were carried out only on trials with correct responses (*n* = 470, 3.4% rejected trials), resulting in a dataset of 11570 observations (i.e., 90% of the original number of observations).

Also in this case, the percentage of wrong answers was homogeneous among Down (3.2%) and Control (4.6%) groups. Logarithmic transformations were applied to correct for positively skewed distributions of accuracy and reaction time scores. All analyses were conducted both with transformed and untransformed values. Because results did not differ, untransformed values are reported for ease of interpretation.

Detailed descriptive statistics of Accuracy and Reaction Times as a function of independent variables are presented in Tables 1, 2. The mean RTs of correct responses and the accuracy proportions were submitted to analyses of variance (ANOVAs) with Prime-Target Congruency (congruent, incongruent), Presentation Type (face-face, face-word, word-face, word-word), Emotion (sad, happy, neutral), as within-subject factors and Group (DS, TD) as between-subject factor. In addition, in order to investigate the interaction between the priming effect and emotional valences more deeply the mean RTs of correct responses and the accuracy proportions were submitted to ANOVAs with Presentation Type (face-face, face-word, word-face, word-word), Prime Emotion (sad, happy, neutral), and Target Emotion (sad, happy, neutral) as within-subject factors and Group (DS, TD) as between-subject factor.

**TABLE 1:** Descriptive statistics: Mean proportions of accuracy and standard errors by Group, Prime-Target congruency, Presentation type and Emotion (*n*_*subjects*_ = 40).

			Group	
Prime-target	Presentation		DS (*n* = 20)	TD (*n* = 20)
congruency	type	Emotion	Mean (SE)	Mean (SE)
Congruent		Sad	0.84 (0.08)	0.93 (0.06)
	Face – face	Happy	0.99 (0.02)	0.98 (0.03)
		Neutral	0.97 (0.04)	0.96 (0.04)
	Face – word	Sad	0.98 (0.03)	0.98 (0.03)
		Happy	0.97 (0.04)	0.98 (0.03)
		Neutral	0.98 (0.03)	0.98 (0.03)
	Word – face	Sad	0.92 (0.06)	0.94 (0.05)
		Happy	0.98 (0.03)	0.98 (0.03)
		Neutral	0.99 (0.03)	0.98 (0.03)
	Word – word	Sad	0.96 (0.04)	0.97 (0.04)
		Happy	0.96 (0.04)	0.97 (0.04)
		Neutral	0.98 (0.03)	0.99 (0.03)
Incongruent	Face – face	Sad	0.81 (0.09)	0.90 (0.07)
		Happy	0.94 (0.05)	0.99 (0.02)
		Neutral	0.94 (0.05)	0.95 (0.05)
	Face – word	Sad	0.99 (0.03)	0.98 (0.03)
		Happy	0.97 (0.04)	0.96 (0.04)
		Neutral	0.97 (0.04)	0.96 (0.05)
	Word – face	Sad	0.86 (0.08)	0.93 (0.06)
		Happy	0.97 (0.04)	0.98 (0.03)
		Neutral	0.92 (0.06)	0.97 (0.04)
	Word – word	Sad	0.90 (0.07)	0.93 (0.06)
		Happy	0.96 (0.04)	0.98 (0.03)
		Neutral	0.98 (0.03)	0.98 (0.03)

**TABLE 2:** Descriptive statistics: Median and mean reaction times (ms) and standard errors by Group, Prime-Target congruency, Presentation type and Emotion (*n*_*subjects*_ = 40).

			Group	
Prime-target	Presentation		DS (*n* = 20)	TD (*n* = 20)
congruency	type	Emotion	Mean (SE)	Mean (SE)
Congruent		Sad	1318 (94)	1592 (91)
	Face – face	Happy	1133 (90)	1235 (58)
		Neutral	1227 (77)	1478 (73)
	Face – word	Sad	1115 (110)	1507 (105)
		Happy	1077 (68)	1318 (94)
		Neutral	1084 (81)	1317 (97)
	Word – face	Sad	1273 (107)	1555 (88)
		Happy	1149 (104)	1222 (66)
		Neutral	1199 (93)	1414 (73)
	Word – word	Sad	1228 (92)	1605 (97)
		Happy	1285 (101)	1431 (85)
		Neutral	1124 (80)	1332 (76)
Incongruent	Face – face	Sad	1430 (101)	1674 (88)
		Happy	1151 (62)	1321 (64)
		Neutral	1297 (91)	1531 (77)
	Face – word	Sad	1119 (95)	1526 (126)
		Happy	1188 (81)	1388 (108)
		Neutral	1104 (84)	1318 (93)
	Word – face	Sad	1465 (125)	1616 (82)
		Happy	1246 (99)	1293 (75)
		Neutral	1311 (98)	1540 (91)
	Word – word	Sad	1281 (92)	1693 (86)
		Happy	1266 (108)	1470 (92)
		Neutral	1136 (86)	1405 (84)

When appropriate, degrees of freedom were adjusted according to the method of Greenhouse-Geisser; only corrected significance levels are reported. The level of significance testing was *p* = 0.05. As *post hoc* mean comparisons (Bonferroni) were employed to further examine significant effects (using a *p* < 0.05 criterion for significance).

## Results

All participants, both with TD and DS did not show any problem performing the priming task and everyone appeared to have understood the instructions.

### Accuracy

For the sake of transparency, in Table 1 descriptive statistics of Accuracy (Mean = 0.96, SE = 0.03) by levels of Group, Congruency, Presentation Type and Emotion are shown. Overall, all participants resulted accurate with an average accuracy well over.85.

**FIGURE 3:**
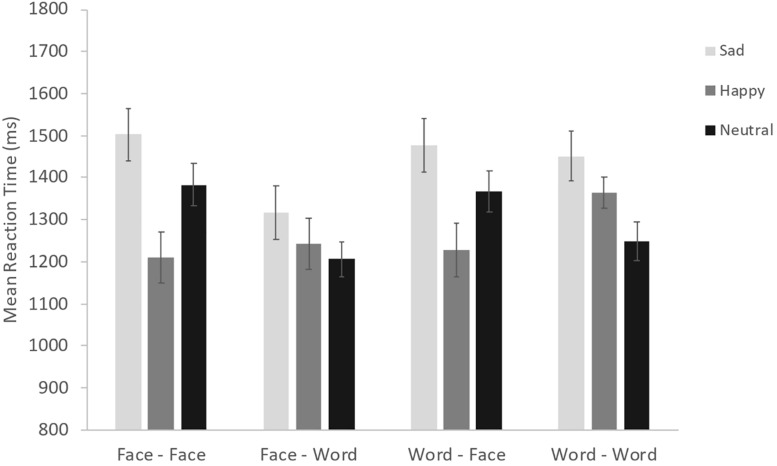
Estimated mean proportions of Accuracy by Presentation Type and Emotion. Error bars represent 95% confidence intervals.

The ANOVA conducted on the accuracy data yielded a significant main effect of Congruency [*F*(1, 38) = 9.52, *p* < 0.01, η_*p^2*_ = 0.20], indicating that participants showed higher accuracy in the congruent condition (Mean = 0.965, SE = 0.006) compared to the incongruent condition (Mean = 0.947, SE = 0.006). However, congruency does not interact with any main factor. On the other hand, a statistically significant interaction between Presentation Type and Emotion was found [*F*(2.83, 107.44) = 5.6, *p* < 0.01, η_*p^2*_ = 0.13; see Figure 3]. *Post hoc* analyses revealed that there are no differences in the accuracy of the recognition of the happy and neutral emotion across all the four conditions: for both, participants resulted highly accurate across all the conditions. The sadness was recognized with less accuracy in all the conditions except in the face-word one, in particular when the target is the face (regardless of the prime) and in the condition word-word (all ps < 0.01). No difficulties were observed in the accuracy of the recognition of the sad emotion in the face-word condition. The second ANOVA, conducted in order to provide a more in-depth investigation into the interaction between the priming effect and emotional valences, did yield a marginally significant Prime Emotion × Target Emotion interaction [*F*(2,56, 97.21) = 2.5, *p* < 0.07, η_*p^2*_ = 0.06]. *Post hoc* analyses revealed a priming effect for all three emotions: grater target accuracy when Prime and Target stimuli shared the same emotion (e.g., sad-sad) than when shared different emotions (e.g., happy-sad; neutral-sad) (all ps < 0.01).

### Reaction Times

In Table 2 descriptive statistics of Reaction Times (Median = 1161.5, Mean = 1307.4, SE = 49.01) by levels of Group, Congruency, Presentation Type and Emotion are shown.

**FIGURE 4:**
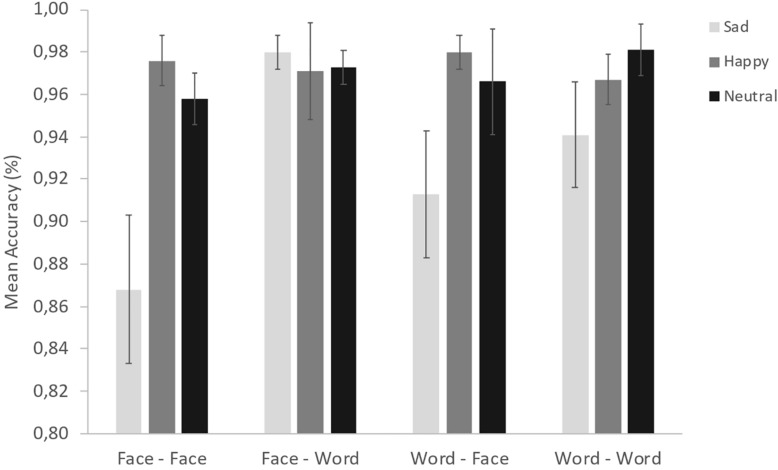
Estimated means of Reaction Time by Group and Emotion. Error bars represent 95% confidence intervals.

**FIGURE 5:**
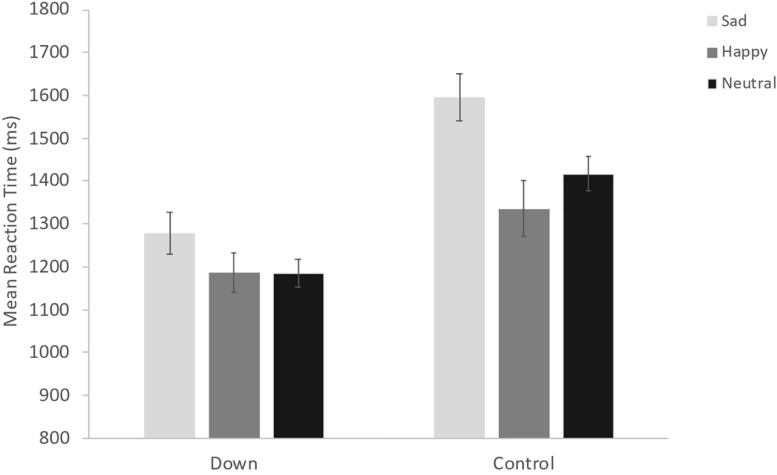
Estimated means of Reaction Time by Presentation Type and Emotion. Error bars represent 95% confidence intervals.

The ANOVA conducted on reaction times showed a significant main effect of Congruency [*F*(1, 38) = 22.86, *p* < 0.001, η_*p^2*_ = 0.38], indicating that participants showed faster RTs in the congruent condition (Mean = 1301, SE = 48.54) compared to the incongruent condition (Mean = 1365, SE = 48.54). However, congruency does not interact with any main factor. We also found a significant Group × Emotion interaction [*F*(1.9, 72.32) = 7.18, *p* < 0.01, η_*p^2*_ = 0.16; see Figure 4], suggesting that individuals with DS are faster than TD children in the recognition of neutral and sadness emotions; the difference between the two groups appear higher for the sadness. Also, in each of the two groups there are differences in reaction times during the recognition of the three emotions: TD children differ in the recognition of all the three categories with slower reaction times for the sadness emotion and faster for the happy emotion; individuals with DS are significantly slower in identifying sadness compared to neutral and happy emotions, but do not show different reaction times in the recognition of the happy and neutral category (all ps < 0.01). Moreover, we found a Presentation Type × Emotion interaction [*F*(4.73, 179.61) = 9.49, *p* < 0.001, η_*p^2*_ = 0.20; see Figure 5]. *Post hoc* analyses revealed that participants are slower in identifying the sadness and the neutral category than the happy one. The recognition of both sadness and neutral categories is slower when they have to identify the emotion through faces, regardless of the prime. The results found that the reaction times for the recognition of the happy emotion were the fastest and did not differ across the presentation type (all ps < 0.01). Interestingly, the second ANOVA, conducted in order to more deeply investigate the interaction between the priming effect and emotional valences, yielded a significant Prime Emotion × Target Emotion interaction [*F*(3.1, 117.62) = 4.46, *p* < 0.01, η_*p^2*_ = 0.11]. *Post hoc* analyses revealed a priming effect for all three emotions: thus faster target RTs when Prime Emotion and Target Emotion stimuli shared the same emotion (e.g., sad-sad) than when they represented different emotions (e.g., happy-sad; neutral-sad) (all ps < 0.01).

## Discussion

The current study was aimed to analyse the ability of adults with DS to identify two basic emotions, namely happiness and sadness in relation to neutrality, either through faces or written words. Individuals with DS were compared to a group of TD children matched for both verbal and non-verbal skills. The research design adopted a priming paradigm, which, to the best of our knowledge was adopted for the first time within this field. For this reason, the current work is preliminary in nature: predictions were not formulated due to an absence of any theoretical account to which to refer to; the results are interpreted as a starting point on which to build our future knowledge regarding the processes underlying emotion processing in DS through a priming paradigm. Nonetheless, the current findings are discussed in relation to the literature concerning emotion recognition and processing in DS in which different experimental and testing paradigms were adopted. The current work provides a contribution in building knowledge on emotion identification in DS, a field which is still open to debate; what is still missing is a clear indication of whether individuals with DS have a specific impairment in emotion recognition or alternatively whether their ability to identify emotions is in line with their cognitive and linguistic level.

The advantage of priming, with respect to other paradigms, is that it allows an investigation into the effect of a particular prime-target relationship without participants’ awareness of the manipulation, such that they cannot develop response strategies. In this case the relation concerned the early identification of an emotion and the distinction of this from neutrality, both through a face expression or a written word. This allowed us to investigate both the ability of participants to distinguish between emotions and neutral conditions and between their ability to identify the target through face and word processing. Another advantage of a priming paradigm is that both accuracy and speed of processing are coded and analyzed.

The findings on accuracy indicated that, in general, participants of both groups reported high accuracy in their answers suggesting that the task was understandable and appropriate for both groups. More importantly for the priming paradigm, the congruency effect emerged significant indicating that participants were less accurate in the incongruent condition than in the congruent one: this result suggests that the priming is effective and the task is appropriate. However, congruency did not interact with other factors as far as accuracy is concerned. Moreover, we failed to find effects of group (both the main effect and the interactions are not significant) in the accuracy during the participant’s performance: perhaps this indicated that the task difficulty is similar for the two groups when matched for verbal and non-verbal skills. As far as the interactions are concerned, a significant interaction between presentation type and emotion emerged. The sadness was identified with less accuracy in all of the conditions except in the condition face-word. Finally, no differences emerged between the identification of the happy and neutral one: for both, participants resulted highly accurate across all the presentation types. A further analysis investigated the interaction between the prime and the target emotion effect: a significant interaction showed that participants of both groups were more accurate in target recognition when prime and target shared the same emotion than when they represented different emotions.

In a priming paradigm, alongside the accuracy of the performance, much more informative is the speed of the performance, since it reveals the underlying processes during the performance. The significance of the main effects is relevant for providing validity to the paradigm: since this is the first time that such a paradigm is adopted in this population and with this material, the main effects are discussed for their preliminary indication of validity. In particular, the main effect of group and congruency provide an indication that the paradigm is appropriate for the target group. Participants provide faster responses in the congruent than in the incongruent condition indicating a priming effect. Individuals with DS resulted generally faster than TD children. Interestingly the two groups do not differ in accuracy.

Furthermore, the factor group interacts with the emotion one. Individuals with DS are particularly fast with respect to TD in the identification of sadness and the neutrality. The two groups did not differ in the identification of happiness. If we compare these results with the accuracy we can note that participants are slow in recognizing the sadness (in particular DS individuals) but at the same time they are less accurate for this category.

Furthermore, the interaction emotion × presentation type indicated that participants are slower when they have to identify sadness in particular through faces. This is less evident when the target is a word. Future studies will have to explore more deeply this result and provide stronger evidence suggesting that more time is needed for processing a face than a word (with the same meaning). Our results are preliminary in nature and future studies will have to replicate the findings by addressing this specific hypothesis. An interesting result emerged concerning the processing of the happy emotion: the reaction times for the recognition of this emotion are faster than for the other two categories, and additionally, the speed of the recognition of the happy emotion does not differ across the four presentations. This means, in other words, that the recognition of the happy emotion is the easiest one, independently of the modality of the presentation (face or word). Furthermore, an additional analysis revealed a significant interaction between prime and target emotion presentation: target RTs were faster when prime and target shared the same emotion than when they represented different emotions.

In the current study, although preliminary, we failed to find specific difficulty in emotion identification for DS: they tend to be faster than TD matched for verbal and non-verbal skills and the two groups show similar levels of accuracy. Other research studies have found evidence of difficulties in emotional understanding in Down syndrome (e.g., Kasari et al., 2001; Williams et al., 2005; Wishart et al., 2007). Our findings that the identification of emotions, both through faces and words, did not represent an area of difficulty beyond what would be expected given their level of verbal and non-verbal cognitive level, is very encouraging. Indeed, except in specific conditions, the performance of individuals with DS and of TD was overall marked more by similarity than difference. This pattern is coherent with some previous results (e.g., Pochon and Declercq, 2013; Channell et al., 2014). The reasons for the discrepancy in findings across different studies are still unclear, but are reasonably related to differences concerning participants characteristics, stimuli type and tasks.

In relation to specific emotions, we found some atypical results for the processing of sadness. Previous literature reported repeatedly difficulties in the processing of fear (e.g., also Wishart et al., 2007). As previously discussed, for the first time, sadness resulted to be processed, in specific circumstances differently than the happy and the neutral categories, especially when the target was a face. Namely, sadness is processed less accurately, more slowly and with a larger priming effect. This result was not different for the two groups. However, differences between the groups were found concerning the speed of processing of the three emotions: individuals with DS were faster than TD in recognizing sadness. Previous works have suggested that the differences between TD and DS can be attributed to qualitative differences in the processing of emotions that may be related to different life experiences with different emotions (e.g., Cebula et al., 2010). In fact, it may be speculated that TD children have less experience with sadness, in general, than with happiness and neutral states given that they were much younger. On the other hand, it is likely that individuals with DS, who are young adults, have had more experience with emotion recognition and in particular have had more opportunities to encounter sadness during their life. Future studies are needed in order to directly address this issue by verifying the ability to recognize sadness in individuals with DS of different ages.

Finally, our research design allowed us to compare participants in the processing of the same stimuli conveyed by faces and by words. It resulted that the two conditions in which the face was the target were more difficult to process than the two conditions with words as targets. This may be related to more basic face processing difficulties highlighted for individuals with DS.

In conclusion, this study provides some preliminary evidence for the effectiveness of the adoption of a priming paradigm with individuals with DS for the investigation of emotion processing. Future studies will have to enhance our knowledge of emotional competencies in children and adolescents with DS by providing more concrete contribution to a better understanding of the emotional competence of people with DS. The present paradigm is very powerful for demonstrating emotion recognition from very early stages of processing. Future studies will have to investigate how these processes relate to emotion knowledge in real-life interactions and how these skills generalize across different social settings. In fact, real-life emotion recognition is much more complex than the controlled setting and selected stimuli presented in the current work. Furthermore, further studies are needed in order to analyze the extent to which individuals with DS are able to use the knowledge on emotions in order to regulate their behavior and language in function of this awareness. Finally, besides investigating the recognition of basic emotions (happy and sad), as in the current work, complex emotions should be included in future studies. All of these aspects are essential in order to better target educational and psychological interventions targeting emotion knowledge for people with DS.

## Data Availability Statement

The datasets for this manuscript are not available publicly since they include sensible participant’s data. The data are kept in the lab by the authors of the paper. Requests to access the datasets should be directed to the corresponding author.

## Ethics Statement

This study was carried out in accordance with the recommendations of the “Italian Association of Psychology” (AIP) Ethical Guidelines (Codice Etico: www.aipass.org/node/11560), was reviewed and received a formal approval by the local Ethical Committe of the Host institution of the first and the third authors (School of Psychology of the University of Padua-Italy). Participants were informed of their rights and gave written informed consent for participation in the study (for children, this consent was granted by their parents), according to the Declaration of Helsinki. All study procedures met the ethical guidelines for protection of human participants, including adherence to the legal requirements of the Country.

## Author Contributions

All authors designed and conceptualized the study, methods, data collection, coded and analyzed the data, wrote the manuscript, and approved the final manuscript.

## Conflict of Interest

The authors declare that the research was conducted in the absence of any commercial or financial relationships that could be construed as a potential conflict of interest.

## Footnotes

1 The faces reported in Figures 1, 2 are not the ones used in the experiment. We chose the image for which a written consent for publication was provided.
